# Diabetic foot ulcer outcomes from a podiatry led tertiary service in Kuwait

**DOI:** 10.1080/2000625X.2018.1471927

**Published:** 2018-05-28

**Authors:** Grace Messenger, Richard Masoetsa, Imtiaz Hussain, Sriraman Devarajan, Mohamed Jahromi

**Affiliations:** a Podiatry Department, Medical Division, Dasman Diabetes Institute, Dasman, Kuwait; b Dasman National Biobank, Research Division, Dasman Diabetes Institute, Dasman, Kuwait; c Clinical Research Unit, Medical Division, Dasman Diabetes Institute, Dasman, Kuwait

**Keywords:** Diabetic foot ulcer, outcomes, risk factors, days to heal, multidisciplinary team, podiatry, diabetes mellitus

## Abstract

**Objective**: This single-centred study aims to evaluate the incidence, risk factors and treatment outcomes of a podiatry led, evidence-based diabetic foot ulcer (DFU) clinic.

**Research design and methods**: Data from the DFU database and patient electronic health records were retrospectively collected from patients with new DFUs who were referred for treatment to the Department of Podiatry, Dasman Diabetes Institute, Kuwait, from 1 October 2014, to 31 December 2016. Patients were followed-up until healing occurred or until 6 months after the study end date, whichever came first.

**Results**: All data were analysed using IBM SPSS version 24 software. Data were collected from 230 patients with 335 DFUs. Most DFUs (67%) were present for <3 months from the time of the first podiatry appointment. A total of 56% of DFUs were classified as neuropathic. Most (72%) DFUs healed, with a median healing time of 52.0 days. Chronic kidney disease (*p *= 0.001), retinopathy (*p *= 0.03), smoking (*p *= 0.02), ulcer location (*p *= 0.03), peripheral arterial disease (PAD) (*p *= 0.004) and osteomyelitis (*p *= 0.05) were found to have a meaningful association with DFU outcome. The number of days to heal was associated with ulcer classification (*p *= 0.005), bacterial infection (*p *= 0.002), osteomyelitis (*p *= < 0.001) and PAD (*p *= < 0.001).

**Conclusions**: The incidence of new DFUs in our tertiary clinic is 3.4%. The incidence of diabetic foot ulceration, days to heal, healing rate and the risk factors influencing healing are in accordance with other multidisciplinary facilities with podiatry input.

## Introduction

Diabetic foot ulcers (DFUs) continue to be a leading cause of non-traumatic lower limb amputation [1], with an estimated lifetime risk of 12–25% [2–4]. The pathway to DFU development is multifactorial, involving several risk factors, including the diagnosis of diabetic peripheral neuropathy (DPN), peripheral arterial disease (PAD), prior DFU and amputation, age, history of diabetic retinopathy suboptimal diabetes control, nephropathy, and end-stage renal disease (ESRD) [4–6]

PAD is associated with poor DFU outcome. The EURODALE multicentre study reported PAD in 52.5% of DFUs, and a significantly reduced healing rate with PAD than without (69% vs. 84%, respectively)[5]. Diabetic foot infection (DFI) reportedly occurs in 40.8–58% of DFU cases[5,7] and osteomyelitis in approximately 20% of DFU, with this figure increasing to over 60% in the presence of severe DFI [8]. Furthermore, PAD and DFI have been associated with some of the most adverse outcomes of DFU and can lead to major amputation [9].

DFUs are categorized into three groups: neuropathic, neuroischaemic and ischemic [10]. Generally, neuropathic DFUs are the largest group accounting for 54% of cases [11]. However, there has been a recent shift, with neuro-ischemic classification overtaking neuropathic to become the largest group (50% vs. 35%, respectively), although, most cases (85%) are complicated by neuropathy [12].

The use of multidisciplinary teams for DFU management is associated with improved outcomes and is now endorsed by most international guidelines [13,14]. Podiatrists should, along with Vascular Surgeons and Diabetologist, be at the forefront of these multidisciplinary teams to provide evidenced-based DFU management [14,15]. However, sizable differences in DFU healing rates (60–77%) and in the median number days to heal (78–241 days) have been reported [7,16–19].

Currently, the incidence, outcome and days to heal of DFUs are used as clinical benchmarks. However, only a few studies have investigated these DFU markers in Arab countries. A recent systematic review identified nine papers from five countries with varying DFU prevalence rates of between 2.7% in Iraq and 11.85% in Saudi Arabia [20]. One paper from Saudi Arabia reported an incidence rate of 1.8% [21]. To date, there are no figures published from Kuwait. Therefore, the present study aimed, to provide data regarding DFU incidence, outcomes and days to heal from the Dasman Diabetes Institute (DDI) as an additional benchmark for multidisciplinary teams in Kuwait and the surrounding region.

For this study, a DFU was defined as a full-thickness wound occurring below the ankle, regardless of the duration, which is associated with DPN and/or PAD in a patient with diabetes based on recommendations of the International Consensus on the Diabetic Foot [13]. Healing was defined as complete epithelialization, as judged by the treating podiatrist.

DPN was defined as the inability of a patient to detect the 10 g monofilament at one or more sites tested [22]. A recent systematic review by Wang et al. (2017) [23] has demonstrated that the use of 10 g monofilament was accurate in diagnosing DPN in patients with diabetes mellitus when compared to Nerve conduction studies. This systematic review showed a pooled sensitivity of 0.53 (95% confidence interval (CI) 0.32–0.74) and a pooled specificity of 0.88 (95% CI 0.78–0.94). In addition, the 10 g monofilament has been found to have a similar diagnostic rate when compared to the 128 Hz tuning fork (32.6 vs 31.4%, respectively). Furthermore, the efficacy of the 10 g monofilament has been established in multiple studies [3,6]. PAD was defined as monophasic Doppler ultrasound readings, an ankle brachial pressure index (ABPI) of <0.9 or the existence of symptomatic vascular disease [4,24].

Microbiology samples were collected if there was a suspicion of soft tissue or bone infection, samples were also collected from DFUs for new patients entering the facility. Samples consisted of surface swabs, deep tissue samples or if possible bone samples. The podiatrists’ considered active infection if there were ≥2 signs of infection (redness, warmth, swelling, induration, pain or tenderness) as defined by the Infectious Diseases Society of America guidelines [25]. Oral or parenteral antibiotic therapy was prescribed on these occasions.

Prior to 2016, Wagner and University of Texas (UT) wound classification systems were arbitrarily used, rendering it difficult to assess DFU grade. Therefore, DFU classification was based on the underlying aetiology; defined as neuropathic, neuroischaemic or ischemic. Meanwhile, the depth of a DFU and presence of osteomyelitis were assessed, and documented as either probe-to-bone (PTB) positive or negative. PTB has been found to have good reliability and sensitivity for the diagnosis of osteomyelitis in this population. A recent systematic review [26], comparing PTB with magnetic resonance imaging, bone histopathology and bone culture, found a pooled sensitivity of 0.87 (95% CI, 0.75–0.93) and a pooled specificity of 0.83 (CI, 0.65–0.93) in seven studies.

DFUs were recorded as healed or not healed. A ‘not healed’ DFU relates to patients either receiving ongoing DFU management, referred to another healthcare provider, lost to follow-up or died.

## Material and methods

The study protocol was approved by the Institutional Research and Ethical Committee of DDI (approval no. RA HM-2017-006).

DDI is a tertiary specialist out-patient facility in Kuwait. DDI provides diabetes management to prevent, mitigate and control diabetes and its complications to a predominantly urban Arab population. Between 1 October 2014 and end of December 2016 a total of 9500 patients attend for at least one specialist clinical appointment.

The Podiatry department is managed by a team of three podiatrists who hold daily clinics. Patients referred with a DFU are treated in accordance with best practice recommendations by the International Working Group on the Diabetic Foot [27]. Treatment depended on the type of ulcer. Most ulcers were treated at a maximum of weekly intervals for evaluation, sharp debridement and review of off-loading. All DFUs were dressed with a suitable dressing for the type of ulcer taking into account levels of exudate, and need for autolytic debridement, with redressing’s either being performed at the patient’s home or local clinic. Neuropathic plantar DFUs were off-loaded using a variety of methods including total contact casts, removable below knee walkers and pressure relieving wound care sandals. Management of neuroischaemic and ischaemic ulcers was in collaboration with the patient’s vascular surgeon; patients were referred to a vascular surgeon if they did not already have one. If patients attended with a severe infection extensive osteomyelitis or soft tissue damage, or sinus track they were referred for parenteral antibiotic therapy and surgical opinion [28]. Patients are referred to an acute hospital setting for the management of limb threating infections, acute osteomyelitis or critical limb ischemia. Patients with PAD are managed across settings in conjunction with Consultant Vascular Surgeons, and it is not uncommon for patients to travel to other countries for surgical management.

During the study period, all new DFUs were recorded on a Microsoft Excel spreadsheet (Microsoft Corporation, Redmond, WA, USA). DFUs above the ankle, infection without a break in the skin, blisters, Charcot Arthropathy without DFU and other unrelated skin diseases were excluded from data analysis.

Patient and DFU demographic information including gender, age, approximate duration of DFU at the time of referral and DFU location were added to the spreadsheet at the patient’s initial appointment. Doppler ultrasound pulse, ABPI, loss of pain sensation, previous DFU-related complications, days to heal and outcome, were added retrospectively. Medical history including the type and approximate duration of diabetes, history of acute vascular event (AVE) including myocardial infarction, cerebrovascular accident and transient ischaemic attacks, pre-existing comorbidities, previous cardiovascular and peripheral vascular intervention and clinical parameters including glycosylated haemoglobin (HbA1c), haemoglobin (Hb) and body mass index (BMI) were also added retrospectively. The duration of diabetes and DFU were recorded as the best guess from patients as most of the study population attended several medical facilities, and there is no central record.

In the event that patients had more than one episode of DFU during the study period, patient demographics, medical history and clinical parameters were only recorded once unless a change was documented in the electronic health record.

## Results

The central tendencies of mean, standard deviation (SD), CIs and median of continuous variables and descriptive statistics for categorical variables were calculated. The relationships between data were analysed using parametric and non-parametric tests. All data analysis was completed using Microsoft IBM SPSS version 24.

Demographic and clinical characteristics are summarized in Table 1. Two hundred and thirty (230) patients with one or more DFU attended during the study period, with a total of 335 new DFUs documented. The mean patient age, HbA1c and BMI at the time of their first podiatry appointment were 62.6 years ± 9.5, 8.8% (73 mmol/mol) ± 2.02 and 33.2 ± 6.7, respectively. A total of 68% of patients were male, 95% were diagnosed with Type 2 diabetes and 59% gave a history of diabetes for greater than 21 years. Previous DFU was recorded in 42% of cases, with previous amputation present in 26%. A previous history of osteomyelitis was present in 26%.

**Table 1.** Clinical characteristic and DFU outcome.

Total sample 230	Frequency (*n*)	Percentage	*p*-Value ulcer outcome	Odds ratio (95% confidence interval)
Total males	156	68	0.1	–
50 years and older	203	88	0.3	–
Type 2 diabetes mellitus	217	95	0.8	–
Diabetes mellitus duration ≥ 20 years.	129	59	0.8	–
Smoker	44	19	0.02^a^	0.23 (0.12–0.45)
History of acute vascular event	26	11	0.6	–
Chronic heart disease	79	34	0.6	–
Chronic kidney disease	58	25	0.001^b^	3.64 (1.38–9.63)
End stage renal disease	20	9	0.01^c^	0.29 (0.11–0.77)
Retinopathy	130	57	0.03^d^	1.77 (0.96–3.28)
Coronary artery revascularisation	44	18	0.4	–
Peripheral artery revascularisation	26	11	0.4	–
History of diabetic foot ulcer	140	61	0.9	–
History of osteomyelitis	62	30	0.9	–
History of amputation	86	37	0.8	–
	Mean	Standard deviation (±)	95% Confidence interval	
Age	62.6	9.5	59.2–65.3	–
Glycosylated haemoglobin (HbA1c)	8.8 (73 mmol/mol)	2.02	8.2–9.3 (66-78 mmol/mol)	–
Body mass index (BMI)	33.2	6.7	31.3–35.0	–
Haemoglobin	122.8	14.6	118.7–126.9	–
Creatinine	129.0	102.7	114.3–158.3	–
Ankle brachial pressure index (ABPI)	1.1	0.4	0.91–1.2	–

*p*-values related to factors which have a significant impact on DFU healing (*p* = 0.05 or less is significant).

^a^Compared to non-smokers and ex-smokers.

^b^Compared to normal kidney function.

^c^Compared to normal kidney function and CKD.

^d^Compared to no proliferation/macular oedema.

Of the 335 newly diagnosed DFUs, 242 (72%) healed during the study period with a median number of days to heal of 52 days (range, 4–573 days). A total of 3% of patients had an ongoing DFU at time of data collection. A total of 25% of patients were lost to follow-up, of this 12% were referred to a vascular specialist or hospital for intravenous antibiotics or surgical opinion.

Most patients presented with a loss of pain sensation (93%) and biphasic Doppler ultrasound readings (52%). The mean ABPI of the study cohort was 1.1 ± 0.4. Most DFUs were classified as neuropathic (56%), followed by neuroischaemic (35%) and ischemic (0.3%), while for some (8%) cases, the medical records contained no classification data. Microbiology samples were collected from 150 DFUs, of which nearly 40% were positive for bacterial growth. The most common microorganisms were Gram-negatives (16%), and 11 (3%) new cases of Methicillin-resistant *Staphylococcus aureus* (MRSA) were identified. Approximately one-third (38%) of DFUs with positive bacterial growth were healed with conservative management including oral and/or intravenous antibiotic therapy. Almost half (46%) of the patients referred to another healthcare provider had DFU with positive bacterial growth. However, the presence of DFI did not have an effect on overall DFU outcome (*p *= 0.4). New diagnosis of osteomyelitis accounted for 22% of cases. A combination of positive X-ray and PTB test results was found in 18% of DFUs, whereas 4% had only a positive PTB tests. More than half (64%) of patients with one or more signs of osteomyelitis went on to heal (*p *= 0.05, odds ratio (OR) = 0.60) (Table 2).

**Table 2.** DFU clinical characteristics related to outcome.

	Total	Healed			
DFU clinical characteristics	*n*	*n*	%	*p*-Value	Odds ratio (95% confidence interval)
Dorsal diabetic foot ulcer (dorsum, toes and digital amputation sites)	153	118	77	0.03^a^	–
≥2 diabetic foot ulcers	66	46	70	0.6	–
Diabetic foot ulcer duration ≥ 1 month	118	86	73	0.8	–
Loss of pain sensation	218	161	74	0.6	–
Monophasic Doppler signal	74	45	63	0.004^b^	0.43 (0.2–0.8)
Neuroischaemic/ischaemic DFU classification	120	77	64	0.02^c^	0.49 (0.29–0.81)
Positive probe-to-bone	66	41	62	0.02^d^	0.52 (0.29–0.92)
Positive X-ray	60	37	62	0.03^e^	0.67 (0.3–1.5)
Osteomyelitis	90	58	64	0.05^f^	0.60 (0.36–1.01)
Positive bacterial growth	129	90	70	0.4	–
Methicillin-resistant *Staphylococcus aureus*	11	8	73	0.4	–

*p*-value related to clinical factors which may or may not affect DFU healing (*p* = 0.05 or less shows significance).

^a^Compared to plantar and calcaneal ulcers.

^b^Compared to biphasic and triphasic Doppler pulses.

^c^Compared to neuropathic DFU.

^d^Compared to negative probe-to-bone.

^e^Compared to negative radiological findings.

^f^Compared to DFU not complicated by osteomyelitis.

The Chi-Square (*χ* ^2^) test was carried out to identify if correlations between the categorical variables and DFU outcome existed, as shown in Table 1, Table 2. There were correlations between treatment outcomes and history of chronic kidney disease (*p *= 0.001, OR = 3.64), ESRD (*p *= 0.01, OR = 0.29), retinopathy *p *= 0.03, OR = 1.77), smoking (*p *= 0.02, OR = 0.23), calcaneal DFU (*p *= 0.01, OR = 0.39), monophasic Doppler signal (*p *= 0.004, OR = 0.43), DFU classification (*p *= 0.02, OR = 0.49), positive X-ray result (*p *= 0.03, OR = 0.67) and positive PTB (*p *= 0.02, OR = 0.52). No correlation was found between DFU outcome and gender (*p *= 0.1), age group (*p *= 0.3), duration of diabetes (*p *= 0.8), history of AVE (*p *= 0.6) chronic coronary disease (*p *= 0.6), coronary artery revascularization (*p *= 0.4) or peripheral artery revascularization (*p *= 0.4).

**Figure 1.**
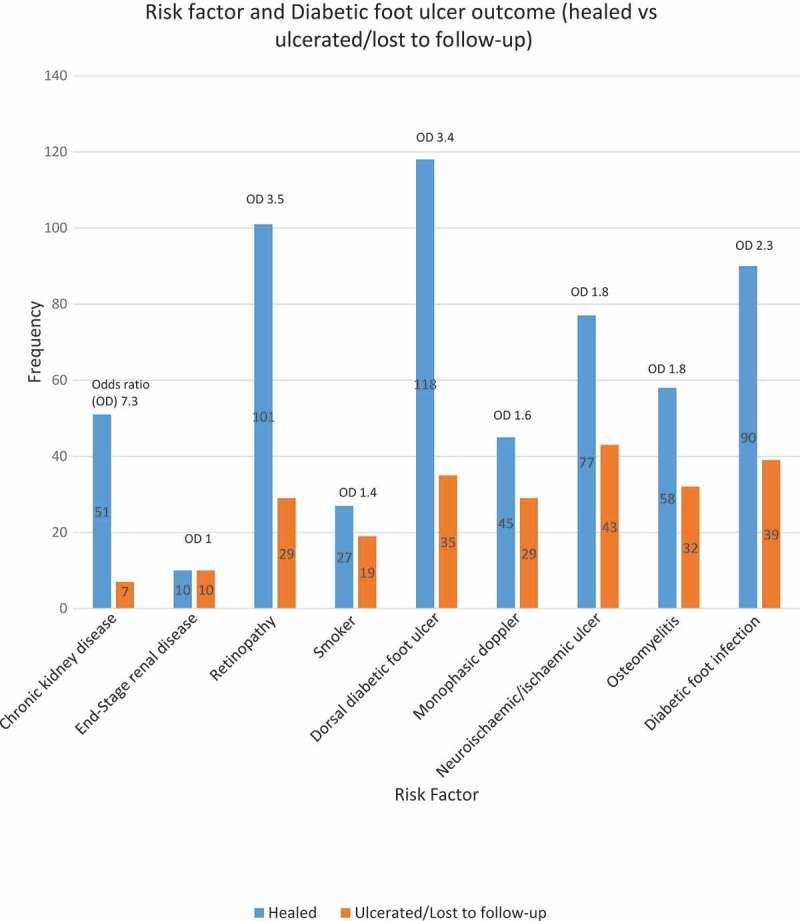
Risk factors and outcome.

One-way analysis of variance revealed that differences in outcome were associated with age (*p *= 0.04), but not with the number of DFUs (*p *= 0.8), the number of comorbidities (*p *= 0.3), the number of vascular interventions (*p *= 0.6), HbA1c (*p *= 0.7), BMI (*p* = 0.06), Hb (*p *= 0.5) and ABPI (*p *= 0.4). The number of days to heal DFU was not normally distributed; therefore, non-parametric tests were performed. As shown in Table 3, the number of days to heal was significantly greater for patients aged <50 than for those aged >50  (38.0 vs. 59.5 days, respectively, *p *= 0.05). DFUs with positive bacterial growth required the greatest number of days to heal compared to those with no bacterial growth or no clinical signs of infection (66.0 vs. 45.0 vs. 43.5 days, respectively, *p *= 0.002). In addition, the type of bacterial growth notably influenced the number of days to heal. MRSA required the greatest number of days to heal and no growth least number of days to heal (100.5 vs. 44.0 days, respectively, *p* ≤ 0.001). DFUs with a positive PTB took longer to heal than those with a negative PTB (77.0 vs. 49.5 days, respectively, *p *= 0.05). The number of days to heal also differed depending on whether X-ray results were positive or negative or if images were not acquired (85.0 vs. 57.0 vs. 44.0 days, respectively, *p *= 0.05). A meaningful difference between days to heal and neuropathic DFU vs. neuroischaemic DFU was observed (47.5 vs. 61 days respectively, *p *= 0.005). There was no difference in median days to heal and gender (*p *= 0.5), duration of diabetes (*p *= 0.4), smoking status (*p *= 0.5), history of AVE (*p *= 0.1), or chronic coronary disease (*p *= 0.1), nephropathy (*p *= 0.8), retinopathy (*p *= 0.3), cardiac revascularization (*p *= 0.1), peripheral revascularization (*p *= 0.2), DFU location (*p *= 0.1), DFU duration (*p *= 0.06) or Doppler signal (*p *= 1.0).

**Table 3.** Days to heal.

Risk factors	Median days to heal	*p*-Value
Male gender	55	0.5
≥50 years age	59.5	0.05^a^
≥20 years diabetes mellitus	53	0.4
Chronic kidney disease	42	0.9
End stage renal disease	72.5	0.3
Proliferative retinopathy	51	0.3
Smoker	62	0.5
History of acute vascular event	75.5	0.1
Chronic cardiac disease	60.5	0.1
Coronary artery revascularisation	64	0.1
Peripheral artery revascularisation	62	0.2
**Diabetic foot ulcer**		
Duration <1 month	43	0.06^b^
Calcaneal location	78	0.1
Neuroischaemic classification	61	0.005^c^
**Clinical investigations**		
Monophasic Doppler signal	62	<0.001^d^
Positive X-ray	83	0.05^e^
Positive probe-to-bone	77	0.05^f^
Total osteomyelitis	77	<0.001^g^
Positive bacterial growth	66	0.002^h^
Methicillin-resistant *Staphylococcus aureus*	100.5	<0.001^i^

Factors that may or may not influence total number of days to heal (*p ≤ *0.05 shows significance).

^a^Compared to patients < 50 years of age.

^b^Compared to DFU present for longer than 1 month.

^c^Compared to neuropathic classification.

^d^Compared to biphasic and triphasic Doppler pulses.

^e^Compared to negative radiological findings.

^f^Compared to negative probe-to-bone.

^g^Compared to DFU not complicated by osteomyelitis.

^h^Compared to no bacterial growth.

^i^Compared to gram-positive, gram-negative growth and no growth.

Ulceration rates at 4, 8, 12 and 24 weeks for patients who continued to attend the department with a DFU remained consistent (3.6%, 3.3%, 4.3% and 3.3%, respectively).

## Discussion

A number of variables associated with outcome and days to heal DFUs were collected in this study, which included patient age, gender, BMI, HbA1c, type and duration of diabetes, pre-existing comorbidities and vascular interventions and smoking status. In addition, multiple variables associated with DFUs were also collected, including DFU duration at first presentation to the podiatry clinic, previous DFU or amputation, previous or current osteomyelitis, and the presence of DPN, PAD or infection. Data were analysed to determine if correlations between variables and complete epithelization and the number of days it took to heal, as judged by a podiatrist existed.

During the study period, the vast majority of patients had type 2 diabetes, with only 5% of patients diagnosed with type 1 diabetes, which is in accordance with diabetes classification for the region around Kuwait [29] and DFU characteristics. The annual incidence of newly diagnosed DFUs is reportedly 2%, which increase to 5–7% in the presence of DPN [30]. In 2011, Margolis [31] reported an incidence rate of 6% among eight hospitals in the USA using Medicare data. The incidence rate of newly diagnosed DFU at DDI is 3.4 cases per 100, which may be associated with the relatively recent manifestation of diabetes in Kuwait. While the age-adjusted comparative prevalence of diabetes exceeds 15% (CI 13.9–18.4) [29], the relatively new appearance of type 2 diabetes in Kuwait may suggest that the full magnitude of long-term complications is yet to appear. In addition, these results were almost certainly affected by the patient cohort recruited from DDI and the level of care received. The patient population attending other specialist clinics in DDI tends to be younger Kuwaitis who are educated to university level and have greater access to intensive diabetes management, which has helped to minimize the number of diabetic foot complications. In addition, the current healthcare systems in Kuwait render it difficult to determine if DFU incidence rates would be higher in other facilities. Furthermore, the incidence of DFU in our institute is almost two-fold greater than that reported in Saudi Arabia of 1.8%[21]. Thus, larger multicentre studies are needed to address the issues raised.

The median of 52 days to heal was similar to the 78 days reported in the UK by Jeffcoate et al. in 2006 [7], where podiatry provision in multidisciplinary settings is well established. This finding is in contrast to the 241 days to heal in India, reported by Viswanathan et al. in 2010 [17]. A possible explanation for these conflicting results is the lack of podiatry services in parts of India [32]. The DFU healing rate in Kuwait is similar to that in the UK and USA (72% vs. 60% and 67.9%, respectively)[7,19].

Although we are currently unable to accurately report amputation rates from this study, a multidisciplinary team approach has been found to reduce the amputation rates by 85%[11]. These outcomes reinforce the importance of a multidisciplinary team comprising of Podiatrists, Diabetologist, Vascular Surgeons and other healthcare professionals for DFU management, including wound debridement, specialist off-loading and infection prevention and management. This study continues to strengthen the debate on the need for podiatry involvement in multidisciplinary clinics.

When compared with previous DFU classification results, the data are similar to those reported by Pecoraro et al. in 1990 [11], with the highest number of DFUs being the neuropathic type (54% vs. 55%, respectively) followed by the neuroischaemic type (34% vs. 36%, respectively). However, the percentage of ischemic DFUs (0.3%) was much lower in this study than reported elsewhere [11,12]. This may be associated with a lower number of patients diagnosed with PAD, as 33% of patients had a monophasic pulse and only 9% of patients had an ABPI of <0.9, and a higher prevalence of DPN, with 93% patients with absent 10 g monofilament response. In addition, 8% of DFUs were not classified or attributed to other factors. In 2016, all podiatrist in DDI began using the UT classification system, and since January 2017, it has become mandatory to grade all DFUs according to NICE guidelines [14] using this classification, to improve the quality of care. Therefore, accurate DFU grading would be available for future studies.

In accordance with the multifactorial pathophysiology of DFU, correlations between DFU outcomes and a number of variables were revealed in this study. Nephropathy, and in particular ESRD, has been strongly correlated to DFU and lower extremity amputation [33]. However, we discovered a positive impact on healing with chronic kidney disease as opposed to its absence (51 vs. 36, respectively, *p *= 0.001, OR = 3.64). In contrast, OR of DFU healing and ESRD was 0.3, compared with patients with no renal disease or those with renal disease without dialysis. Although only a small proportion of patients admitted to smoking, DFU healing was greatly reduced among current smokers (*p *= 0.02, OR = 0.23). Moreover, the presence of PAD and monophasic Doppler signals was correlated with delayed DFU (*p *= 0.004, OR = 0.43). These findings support those of previous studies that ESRD, smoking, and PAD delay DFU healing [12,34]. However, a greater number of DFUs progressed to full epithelization as judged by a podiatrist despite the presence of risk factors as illustrated in Figure 1.

In this study, >20% of patients were diagnosed with osteomyelitis, which is in accordance with a 2006 report by Lipsky et al. 2006 [35], who reported osteomyelitis in 10–20% of DFU cases. Moreover, positive PTB (*p *= 0.02, OR = 0.52) and positive X-ray findings (*p *= 0.03, OR = 0.67) were significantly associates with an increased number of days to heal. Also, there was a significant correlation between DFU outcome and the presence of osteomyelitis (*p *= 0.05, OR = 0.60). These findings are similar to those of other studies and further highlight the destructive nature of DFI and osteomyelitis [25].

Our DFU rates remained relatively consistent at 4, 8, 12 and 24 weeks. However, DFU recurrence rates were difficult to establish accurately as many of the patients were lost to follow-up after referral for parenteral antibiotic therapy and surgical opinion or ulcer healing was achieved. Further research needs to be carried out to establish accurate long-term DFU outcomes.

## Study limitations

The amount of missing or incomplete data was a major limitation to this retrospective cross-sectional analysis, which is a well-recognized limitation in retrospective data collection [36,37]. Accordingly, a number of variables described in this study had a weak or no correlation to DFU outcome or days to heal, despite previous correlations reported. Therefore, future prospective studies are warranted to identify other significant relationships.

During the study period, patients who were referred to another healthcare provider or those who failed to attend their podiatry appointment were not contacted to arrange ongoing appointments or to inquire about treatment outcomes. Thus, the collection of data regarding treatment and DFU outcomes including re-ulceration rates was dependent on the patient contacting DDI to schedule follow-up appointments. However, this system is unreliable, and the burden should be with the healthcare provider to contact patients who fail to attend scheduled appointments rather than on patients, who often have multiple healthcare appointments. In future, we plan to follow patients who fail to attend appointments or who are referred to outside providers in an attempt to provide cohesive care and collect missing data.

Although medically trained translators assist in consultations between non-Arabic speaking clinicians and non-English speaking patients at DDI, this study was further limited by inaccurate or incomplete background data. The language barrier has been associated with poor patient comprehension because the patient may not understand the clinician’s requests, lack the health vocabulary to accurately report symptoms or may recount information in an illogical or fragmented manner [38]. The use of either medically trained or ad hoc translators can reduce the impact of these problems, but do not eliminate them [39].

## Conclusions

To the best of our knowledge, this is the first report on DFU incidence and treatment outcomes in Kuwait and one of a few from this region. These results provide insights into the problems of DFU encountered in Kuwait. However, reporting outcomes of DFU, days to heal and risk factors must continue, to allow benchmarking for healthcare facilities in the region. The data also support the use of international guidelines and multidisciplinary teams in the management of DFU, consistent with previously published DFU outcomes.
